# Expression of stem cell and epithelial-mesenchymal transition markers in primary breast cancer patients with circulating tumor cells

**DOI:** 10.1186/bcr3099

**Published:** 2012-01-20

**Authors:** Sabine Kasimir-Bauer, Oliver Hoffmann, Diethelm Wallwiener, Rainer Kimmig, Tanja Fehm

**Affiliations:** 1 Department of Gynecology and Obstetrics, University Hospital of Essen, University of Duisburg-Essen, D-45122 Essen, Germany; 2 Department of Gynecology and Obstetrics, University Hospital of Tuebingen, D-72076 Tuebingen, Germany

## Abstract

**Introduction:**

The presence of circulating tumor cells (CTC) in breast cancer might be associated with stem cell-like tumor cells which have been suggested to be the active source of metastatic spread in primary tumors. Furthermore, to be able to disseminate and metastasize, CTC must be able to perform epithelial-mesenchymal transition (EMT). We studied the expression of three EMT markers and the stem cell marker ALDH1 in CTC from 502 primary breast cancer patients. Data were correlated with the presence of disseminated tumor cells (DTC) in the bone marrow (BM) and with clinicopathological data of the patients.

**Methods:**

A total of 2 × 5 ml of blood was analyzed for CTC with the *AdnaTest BreastCancer *(AdnaGen AG) for the detection of EpCAM, MUC-1, HER2 and beta-Actin transcripts. The recovered c-DNA was additionally multiplex tested for three EMT markers [TWIST1, Akt2, phosphoinositide kinase-3 (PI3Kα)] and separately for the tumor stem cell marker ALDH1. The identification of EMT markers was considered positive if at least one marker was detected in the sample. Two BM aspirates from all patients were analyzed for DTC by immunocytochemistry using the pan-cytokeratin antibody A45-B/B3.

**Results:**

Ninety-seven percent of 30 healthy donor samples investigated were negative for EMT and 95% for ALDH1 transcripts, respectively. CTC were detected in 97/502 (19%) patients. At least one of the EMT markers was expressed in 29% and ALDH1 was present in 14% of the samples, respectively. Interestingly, 5% of the ALDH1-positive and 18% of the EMT-positive patients were CTC-negative based on the cut-off level determined for CTC-positivity applying the *AdnaTest BreastCancer*. DTC in the BM were detected in 107/502 (21%) patients and no correlation was found between BM status and CTC positivity (*P *= 0.41). The presence of CTC, EMT and ALDH1 expression was not correlated to any of the prognostic clinical markers.

**Conclusions:**

Our data indicate that (1) a subset of primary breast cancer patients shows EMT and stem cell characteristics and (2) the currently used detection methods for CTC are not efficient to identify a subtype of CTC which underwent EMT. (3) The clinical relevance on prognosis and therapy response has to be further evaluated in a prospective trial.

## Introduction

Disseminated tumor cells (DTC) in the bone marrow (BM) and circulating tumor cells (CTC) in blood are suggested as potential surrogate markers for minimal residual disease, the precursor of metastatic disease. Their presence in blood and BM of primary breast cancer patients represents a strong independent prognostic factor for reduced disease-free and overall survival [1-4]. In addition, it has been demonstrated that tumor cells frequently survive chemotherapy [5,6] and that their persistence in BM and blood after conventional adjuvant chemotherapy is associated with poor prognosis [7-9].

One currently discussed hypothesis for the persistence of these cells is the theory that some DTC or CTC are cancer stem cells which have been suggested to be the active source of metastatic spread in primary tumors [10,11]. For DTC, at least one study has confirmed a putative stem cell phenotype showing that CD44^+ ^CD24^- ^cells were detected in the BM of all patients with cytokeratin (CK)-positive cells, whereas their mean prevalence among the total number of DTC was 72% [12].

Among CTC identified in patients with metastatic breast cancer, 32% had the stem cell like phenotype CD44^+^CD24^- ^and 17% of the CTC were positive for one other recently proposed stem cell marker, aldehyde dehydrogenase 1 (ALDH1) [13] which has been associated with poor prognosis in different forms of breast cancer [14,15]. In addition, CTC from patients with primary and metastatic breast cancer were shown to express receptors and activated signaling kinases of the EGFR/HER2/PI3K/Akt pathway [16], which has been described as one of the major pathways in the regulation of mammary stem/progenitor cells, promoting proliferation and the inhibition of apoptosis [17]. We recently demonstrated that CTC in primary breast cancer patients are generally triple-negative [18], which is in concordance with the cancer stem cell theory [19,20].

To be able to disseminate from the primary tumor and metastasize, tumor cells have to undergo phenotypic changes, known as epithelial-mesenchymal transition (EMT), which allows them to travel to the site of metastasis formation without getting affected by conventional treatment. For CTC, EMT and stem cell features have recently been documented in 92 breast cancer patients in all stages of the disease [21]. In metastatic breast cancer, we were able to show that a major proportion of CTC found in the blood of cancer patients shows EMT and tumor stem cell characteristics and that CTC expressing EMT and tumor stem cell markers were an indicator for therapy resistant cell populations and thus, for an inferior prognosis [22].

So far, no data are available for large cohorts of primary breast cancer patients. Assuming that metastasis requires a dissemination of tumor stem cells or tumor cells showing EMT, we studied the expression of (1) the EMT markers Akt2, phosphoinositide kinase-3α (PI3K-α) and the transcription factor TWIST1 and (2) the stem cell marker ALDH1 in CTC isolated from 502 patients with primary breast cancer. Data were correlated with the presence of DTC in the BM and clinicopathological data of the patients.

## Materials and methods

### Patient population and patient characteristics

The study was conducted at the Department of Obstetrics and Gynecology in Essen and in the Department of Obstetrics and Gynecology in Tübingen. In total, 502 primary breast cancer patients (pT1-4, pN0-2, Mo) have been studied. Patients' characteristics at the time of diagnosis are shown in Table 1.

**Table 1:** Clinical data of patients

Characteristics	Total	CTC pos	In %	*P*-value
Total	502	97	19	-
Tumor Size				
pT1	322	58	18	0.32
pT2-4	180	39	22	
Nodal Status				
Negative	340	65	19	0.79
Positive	159	32	20	
Histology				
Ductal	392	78	20	0.83
Lobular	77	13	17	
Others	30	6	20	
Grading				
G I	95	18	19	0.68
G II	290	53	18	
G III	113	25	22	
ER status				
Positive	415	77	19	0.34
Negative	87	20	23	
PR status				
Positive	382	72	19	0.63
Negative	120	25	21	
HER2 status				
Negative	437	83	19	0.50
Positive	62	14	23	
DTC				
Negative	367	69	19	0.41
Positive	107	24	21	
Subtype by IHC				
(ER-, PR-, HER2-)	58	14	24	0.62
(ER-, PR-, HER2+)	17	3	18	
(ER+ and/or PR+)	424	80	19	

### IGROV- cell line

The ovarian cancer cell line was purchased from the ATCC (American Tissue Culture Collection, Rockville, MD, USA) and cultured in a humidified incubator at 37°C in an atmosphere of 5% CO_2 _and 95% air. The cell line was maintained in RPMI medium supplemented with 10% heat-inactivated fetal bovine serum and 1% penicillin/streptavidin (Biochrom KG, Seromed, Berlin, Germany).

### Healthy controls

A total of 5 ml of blood was collected from 30 healthy donors aged 23 to 73 years for the determination of specificity and sensitivity of the applied tests for the determination of ALDH1 and EMT markers.

### Collection and analysis of BM

Between 10 and 20 ml BM were aspirated from the anterior iliac crests of all primary breast cancer patients at the time of surgery and processed within 24 hours. All specimens were obtained after written informed consent and collected using protocols approved by the institutional review board (114/2006A/05/2856). Tumor cell isolation and detection was performed based on the recommendations for standardized tumor cell detection recently published by the German Consensus group of Senology [23]. Details of the staining procedure and cell detection have been described elsewhere [18].

### Sampling of blood

Two × 5 ml EDTA blood was collected for isolation of CTC before the application of therapeutic substances with an S-Monovette^® ^(Sarstedt AG & Co., Nümbrecht, Germany) and stored at 4°C until further examination. The samples were processed immediately or not later than four hours after blood withdrawal. An additional serum sample was collected to determine serum tumor markers.

### Tumor cell enrichment/selection

Blood samples were taken from 502 patients and analyzed for CTC with the *AdnaTest BreastCancer *(AdnaGen AG, Langenhagen, Germany), which enables the immunomagnetic enrichment of tumor cells *via *epithelial and tumor associated antigens. In brief, blood samples were incubated with a ready-to-use antibody mixture (against GA733-2 and MUC-1) commercialized as *AdnaTest BreastCancerSelect *according to the manufacturer's instructions. The labelled cells were extracted by a magnetic particle concentrator (MPC). Subsequently, mRNA isolation from lysed, enriched cells was performed according to the manufacturer's instructions with the Dynabeads mRNA DIRECT^™ ^Micro Kit (Dynal Biotech GmbH, Hamburg, Germany) that is included in the *AdnaTest BreastCancerDetect*. Reverse transcription resulted in cDNA, which was the template for tumor cell detection and characterization by multiplex RT-PCR. Sensiscript^® ^Reverse Transcriptase (QIAGEN GmbH, Hilden, Germany) was used for the reverse transcription because of its high sensitivity (recommended for amounts of < 50 ng RNA) in combination with oligo(dT) coupled Dynabeads of the mRNA DIRECT^™ ^Micro Kit (Dynal Biotech GmbH) according to the manufacturer's instructions [24].

### Tumor cell detection

The *Adnatest BreastCancerDetect *was used for the detection of breast cancer-associated gene expression in immunomagnetically enriched tumor cells by reverse transcription and PCR. The analysis of tumor-associated mRNA isolated from CTC was performed in a multiplex PCR for the three tumor-associated transcripts HER2, MUC-1 and GA 733-2 followed by storage of the samples at 4°C.

The primer sets for the ER and PR receptor were provided from Adnagen^®^AG (Langenhagen, Germany). ER and PR were detected on CTC performed with these reagents after the preparation of the cDNA and according to the instructions of the *AdnaTest BreastCancerDetect*. PCR was performed with the HotStarTaq Master Mix (QIAGEN GmbH). Actin was used as an internal PCR positive control. The thermal profile used for the nested RT-PCR was as follows: After a 15-minute denaturation at 95°C, 37 cycles of PCR were carried out by denaturation at 94°C for 30 s, annealing/extension at 60°C for 30 s and elongation for 30 s at 72°C. Subsequently, termination of the reaction was carried out at 72°C for five minutes followed by storage of the samples at 4°C.

The primers generate fragments of the following sizes: GA 733-2: 395 base pairs (bp), MUC-1: 293 bp, HER2: 270 bp, and actin: 114 bp. Visualization of the PCR fragments was carried out with a 2100 Bioanalyzer using the DNA 1000 LabChips (Agilent Technologies and the Expert Software Package (version B.02.03.SI307) both Böblingen, Germany.

### Evaluation of data established for CTC

The test is considered positive if a PCR fragment of at least one tumor associated transcript (MUC-1, GA 773-2 or HER2) is clearly detected. Using the software package for evaluation of the data on the Agilent 2100 Bioanalyzer, peaks with a concentration of > 0.15 ng/μl are positive for the transcripts GA733-2, MUC-1 and HER2.

### The AdnaTest TumorStemCell/The AdnaTest EMT

Both tests require the enrichment of CTC from 5 ml blood using the *AdnaTest BreastCancerSelect *prior to the singleplex PCR assay to analyze ALDH1 and the multiplex PCR assay to analyze EMT markers and actin as an internal control. Contaminating leukocytes (about 1,500 per sample) are reduced 10-fold by using a special washing procedure (AdnaWash buffer). This enables the proper differentiation of EMT and tumor stem cell expression profiles with a specificity of > 90% which was confirmed in healthy donor samples.

The thermal profile used for EMT multiplex RT-PCR was as follows: After a 15-minute denaturation at 95°C, 35 PCR cycles were carried out by denaturation at 94°C for 30 s, annealing/extension at 60°C for 30 s and elongation for 45 s at 72°C. Subsequently, termination of the reaction was carried out at 72°C for 10 minutes followed by storage of the samples at 4°C. The primers generate fragments of the following sizes: Akt-2: 306 bp, TWIST 1: 203 bp, PI3Kα: 595 bp and Actin: 119 bp, respectively.

The thermal profile used for ALDH1 singleplex PCR was as follows: After a 15-minute denaturation at 95°C, 35 PCR cycles were carried out by denaturation at 94°C for 30 s, annealing/extension at 51°C for 30 s and elongation for 30 s at 72°C. Subsequently, termination of the reaction was carried out at 72°C for five minutes followed by storage of the samples at 4°C. The generated fragment size for ALDH1 is 165 bp.

### Immunohistochemical analysis of the primary tumor

For each of the 502 patients, the tumor type, TNM-staging and grading were assessed according to the WHO-Classification of tumors of the breast [25] and the sixth edition of the TNM Classification System [26]. The ER and PR receptor status were routinely determined by immunohistochemistry in the departments of pathology of each University Hospital. The DAKO-Score for the expression of HER2 was determined with the HercepTest and FISH analysis in cases of 2+ staining as determined with the HercepTest was performed as described elsewhere [27]. Details have been described elsewhere [18].

### Statistical analysis

The chi-squared test or Fisher's exact test was used to evaluate the relationship between DTC and CTC and clinicopathological factors. Statistical analysis was performed by SPSS, version 11.5 (SPSS Inc., Chicago, IL, USA). *P*-values below 0.05 were considered statistically significant.

## Results

### Patients'characteristics

A total of 502 patients were included in the study. Clinical data are shown in detail in Table 1. The majority of patients had tumors smaller than 2 cm and were node-negative. A total of 415 of 502 (82%) patients were ER-positive and 382 of 502 patients were PR-positive, respectively. HER2 expression was present in 62 of 502 patients (12%). Classifying tumors in subtypes based on their ER, PR and HER2 expression, 424/502 (85%) of the tumors were ER and/or PR positive, 58/502 (12%) were triple-negative (ER-/PR-/HER2-) and 17 tumors (3%) only expressed HER2 (ER-/PR-/HER2+).

### Correlation of CTC with established prognostic markers

A blood sample was regarded CTC positive if at least one of the three markers EPCAM, MUC-1 or HER2 was expressed. The overall detection rate for CTC was 19% (97/502 patients). The presence of CTC did not significantly correlate with any of the clinicopathological factors. DTC were found in 107/502 (21%) patients. No correlation could also be observed between positive BM status and CTC positivity (*P *= 0.41; Table 1).

### Correlation of CTC and EMT markers/ALDH1

**Figure 1:**
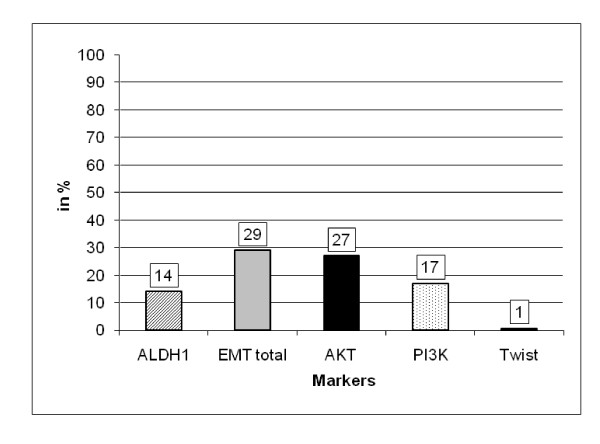
**Expression of EMT-markers and ALDH1**. At least one of the EMT markers was expressed in 29% and ALDH1 was present in 14% of the samples, respectively.

**Figure 2:**
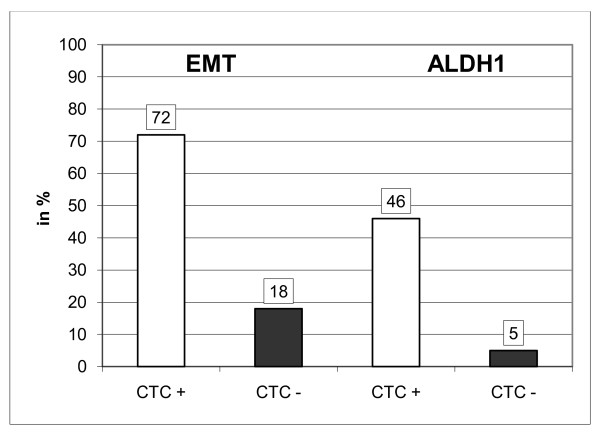
**Expression of EMT-markers and ALDH1 in CTC+ and CTC- patients**. In the CTC^+ ^group, 66 of 92 patients (72%) were positive for at least one of the EMT markers and 42 of 92 patients (46%) were positive for ALDH1, respectively. In the CTC^- ^group, the percentages were 18% (63 of 354 patients) and 5% (19/353 patients).

All samples were further examined for ALDH1 and EMT markers and correlated with the presence of CTC. The complete data set for the detection of CTC, the expression for ALDH1 as well as the EMT markers could be determined in 446/502 patients. At least one of the EMT markers was expressed in 29% (Akt2 27%; PI3K 17%; TWIST 1%) and ALDH1 was present in 14% of the samples, respectively (Figure 1). In the CTC^+ ^group, 66 of 92 patients (72%) were positive for at least one of the EMT markers and 42 of 92 patients (46%) were positive for ALDH1, respectively. In the CTC^- ^group, the percentages were 18% (63 of 354 patients) and 5% (19/353 patients) (Figure 2). Table 2 shows a more detailed analysis of the distribution of the individual markers among the CTC^+ ^and CTC^- ^groups. In the CTC^+ ^group, the expression rates for the EMT markers were 1% (TWIST), 70% (Akt2), and 52% (PI3Kα) and in the CTC^- ^group, the percentages were 1%, 16%, and 8%, respectively (Table 2). Positivity with EMT markers or ALDH1 was not associated with any of the clinicopathological factors.

**Table 2:** Expression of EMT markers and ALDH1 in CTC-positive and CTC- negative patients

	N	EMT	AKT	PI3K	TWIST	N	ALDH1 pos
**CTC neg**	354	63 (18%)	57 (16%)	28 (8%)	2 (1%)	353	19 (5%)
**CTC pos**	92	66 (72%)	64 (70%)	48 (52%)	1 (1%)	92	42 (46%)
**Total**	446	129 (29%)	121 (27%)	76 (17%)	3 (1%)	445	61 (14%)

## Discussion

Enumeration of CTC in breast cancer, using the FDA approved CellSearch technology, has been well established in several clinical studies, showing a correlation with decreased progression-free survival and overall survival in primary as well as in metastatic breast cancer before the initiation of treatment [28-31]. Monitoring studies provided valuable clinical information for the management of breast cancer patients and an ongoing prospective randomized clinical trial is now addressing the question whether to change an existing treatment protocol according to the persistence of CTC after three to five weeks of therapy instead of waiting for clinical and radiologic evidence of progression in metastatic breast cancer patients [SWOG S0500, clinicaltrials.gov, identifier: NCT00382018].

Despite the prognostic impact of CTC counts in breast cancer, mainly using EpCAM-based capturing methods, it has been shown that this procedure is not able to detect the entire, highly heterogenous population of CTC [32,33]. In addition, their biological features are poorly understood and it is assumed that only a few CTC are able to grow into clinically evident metastases [34]. Furthermore, the current belief is that EMT processes, also in the primary tumor, play key roles in the invasion steps of the metastatic cascade involved in the generation of the so called breast cancer stem cells [35].

Taking all these considerations into account, we applied a multimarker RT-PCR assay for the identification of CTC-associated transcripts, using an antibody 'cocktail' for capturing CTC of epithelial origin. This test procedure has been successfully applied for the detection and characterization of CTC in gynaecological cancers and the prognostic value has been shown for monitoring of metastatic breast cancer patients and recently for primary ovarian cancer patients where the detection of CTC before surgery and after platinum-based chemotherapy significantly correlated with a reduced overall survival [18,22,36,37]. Interestingly, a very recent publication demonstrated that the AdnaTest complements the CellSearch system by improving the overall detection rate and permitting the assessment of genomic markers in CTC in metastatic breast cancer patients [38]. Using this technology, we demonstrate here, by the analysis of 502 primary breast cancer patients, that a subset of patients shows EMT and stem cell characteristics and that the currently used detection methods for CTC are not efficient to identify a subtype of CTC which underwent EMT or display a cancer stem cell phenotype. To our knowledge, this is the biggest patient cohort published up to now, analyzing these CTC characteristics.

At least one of the EMT markers was expressed in 29% and ALDH1 was present in 14% of the samples, respectively. Very recently published data in smaller patient groups support our data. Raimondi *et al. *investigated the expression of EMT and stemness markers in 92 breast cancer patients at all stages of the disease. Using the standard definition of CTC as EpCAM+/CK+/CD45-, the authors failed to find them in 31/92 (34%) patients. The absence of CTC was defined as the lack of expression in the enriched EpCAM-positive fraction. Nevertheless, in 12/31 (38%) of these CK-negative cells they found the expression of EMT markers, specifically vimentin and fibronectin. They concluded that current CTC detection methods underestimate the most important subpopulations of CTC involved in cancer dissemination, which often share EMT and stemness features [21]. These data are in accordance with our findings, showing that 5% of the ALDH1-positive and 18% of the EMT-positive patients were CTC-negative based on the cut-off level determined for CTC-positivity applying the *AdnaTest BreastCancer*.

Reuben *et al. *assessed the presence of Aldeflour+epithelial (CD326+CD45dim) cells for the presence of the CD44+CD24lo phenotype in the BM of patients with primary breast cancer. They report that the percentage of CD44+CD24lo cancer stem cells in the BM is higher in primary breast cancer patients with high risk tumor features. Furthermore, patients who received neoadjuvant chemotherapy were more likely to have percentages of Aldeflour+ epithelial cells than the highest level of healthy donors [11].

In a retrospective study, including 292 metastatic breast cancer patients, CTC were enumerated before patients started a new line of treatment using the CellSearch System. CTC were not detected in 36% of the patients. In multivariate analysis, brain metastasis and bone involvement remained independent variables associated with undetectable CTC status. Also, patients with negative hormone receptors, high tumor grade, triple-negative disease and inflammatory breast cancer had increased probabilities for undetectable CTC. The authors suggested that these findings could be due to an under estimation of CTC by CellSearch due partly to CTC undergoing EMT [39]. A second observation of this study was a significant association of the absence of bone metastasis and undetectable CTC status as well.

This again raises the frequently asked question of whether BM analysis for the detection of DTC can be replaced by blood sampling for the detection of CTC. In our present study, as well as in our recently published study, comparing DTC and CTC in 431 primary breast cancer patients, we documented a weak concordance between the presence of these cells in BM and blood, concluding that the biology of these cells may be different [18]. These data are in accordance with other comparison studies where, in general, the correlation between DTC and CTC was shown to be low [40-43]. In brief, the detection of CTC appeared to be less sensitive and less prognostic and the prognosis of women with primary breast cancer depended on DTC rather than CTC. However, two other recently published studies found that the determination of CTC status by RT-PCR conveyed clinically relevant information that was not inferior to DTC status in breast cancer [44,45]. Conclusively, blood analysis can give complementary information to that obtained by BM analysis.

There are different explanations for this phenomenon. 1) The absence of CTC as described above by Mego *et al. *may also be dependent on the inadequacy of CTC standard selection procedures. Most selection procedures are based only on EpCAM, which possibly gets lost during the progress of EMT. 2) EMT cells, degrading the surrounding matrix, also allow the detachment and dissemination of non-EMT tumor cells. Thus, DTC and CTC may reflect the heterogeneous tumor cell population of the primary tumor, including differentiated and more stem cell-like tumor cells. 3) One may also speculate that bone metastasis requires a different mechanism of dissemination or epithelial differentiation.

The PTEN/Akt/mTOR pathway has been described: 1) as one of the major pathways in the regulation of mammary stem/progenitor cells, 2) to confer resistance to conventional therapy, and 3) to play a central role in the viability and maintenance of cancer stem cells in breast, promoting proliferation and the inhibition of apoptosis [17,46]. Furthermore, cells undergoing EMT have been shown to develop resistance to anti-cancer agents [47].

Only a few studies have addressed these aspects in CTC. By analyzing peripheral blood mononuclear cell cytospins from 28 CK-positive and 17 CK-negative patients samples using confocal laser scanning microscopy, phosphorylated PI-3 kinase (phospho PI3K) was documented in 15 of 17 CK- positive samples [48]. In a recent study, the authors analyzed peripheral blood mononuclear cells from 32 cytokeratin-19 mRNA-positive patients with early and 16 patients with metastatic breast cancer and reported that phospho PI3K/Akt were identified in a significant proportion of CTC [16]. These data support the data of this study as well as our recently published data in metastatic breast cancer showing that a major proportion of CTC expressed PI3K, Akt2 as well as the transcription factor TWIST and ALDH1 which was associated with therapy resistance and an inferior prognosis [22].

In summary, the expression of stemness and EMT markers in CTC has been found associated with resistance to conventional anti-cancer therapies and treatment failure, highlighting the urgency of improving tools for detecting and eliminating minimal residual disease [49]. Consequently, the detection of stemness-like cells is highly recommended for therapeutic and diagnostic implications. Signaling pathways that maintain cancer stem cells are attractive targets for these therapies. Preclinically, everolimus (RAD001), an oral inhibitor of mTOR acting downstream of the PI3K/AKT pathway, has been shown to reverse resistance to tamoxifen. In a phase II study, RAD001 combined with tamoxifen provided significant improvement in the six-month clinical benefit rate compared to tamoxifen alone [50]. Furthermore, RAD001 had effective inhibitory effects on cancer stem cells *in vitro *and *in vivo *and, combination treatment with RAD001 and docetaxel or Herceptin^® ^may be effective in refractory metastatic breast cancer [51]. In addition, combinational therapy aimed at Shh and mTOR signaling inhibition, together with standard chemotherapy, proved to be capable of selectively eliminating cancer stem cells [52].

Li *et al. *demonstrated that the remaining tumorigenic cells after chemotherapy had unique properties of enhanced self-renewal as demonstrated by formation of mammospheres and increased propensity for tumor formation. In addition, lapatinib did not lead to an increase in these tumorigenic cells, and thus, in combination with conventional therapy, specific pathway inhibitors may provide a therapeutic strategy for eliminating these cells to decrease recurrence and improve long-term survival [53]. Another promising agent is salinomycin where global gene expression analyses showed that salinomycin treatment resulted in the loss of expression of breast cancer stem cells [54].

## Conclusions

The cancer stem cell hypothesis is a new paradigm that could have a major impact on the treatment of disease by suggesting a new target for cancer therapy. Current treatments of cancer have shown efficacy in removing the bulk of differentiated cancer cells while failing to eliminate the cancer stem cells responsible for tumor relapse. Targeting residual cells with stem cell self-renewal properties in combination with conventional chemotherapy may provide a specific approach to prevent cancer recurrence and improve long-term survival.

## Abbreviations

AdnaGen AG: *AdnaTest BreastCancer *; AdnaGen is the company: AdnaTest BreastCancer is a commercially available; CTC test ATCC: American Tissue Culture Collection; BM: bone marrow; CK: cytokeratin; CTC: circulating tumor cells; DTC: disseminated tumor cells; EMT: epithelial-mesenchymal transition; MPC: magnetic particle concentrator; PI3Kα: phosphoinositide kinase-3α.

## Competing interests

The authors declare that they have no competing interests.

## Authors' contributions

SKB, OH and TF made substantial contributions to the conception and design of the study, acquisition of data, analysis and interpretation of the data. TF and SKB were involved in drafting the manuscript or revising it. All authors read and approved the final manuscript.
